# Potassium‐Competitive Acid Blocker‐Associated Gastric Hyperplastic Polyps With Severe Bleeding: A Case Series

**DOI:** 10.1002/deo2.70326

**Published:** 2026-04-14

**Authors:** Kengo Kasuga, Sakuya Katakai, Megumi Shimizu, Ayaki Isshiki, Shingo Ishihara, Hiroomi Ogawa, Xing Hua Ma, Takashige Masuo, Yoji Takeuchi, Toshio Uraoka

**Affiliations:** ^1^ Department of Gastroenterology Isesaki Municipal Hospital Gunma Japan; ^2^ Department of Gastroenterology and Hepatology Gunma University Graduate School of Medicine Gunma Japan; ^3^ Department of Surgery Isesaki Municipal Hospital Gunma Japan

**Keywords:** acid suppression therapy, gastrointestinal hemorrhage, histamine H2 antagonists, hypergastrinemia, Vonoprazan

## Abstract

Gastric hyperplastic polyps can cause gastrointestinal bleeding, and their enlargement has been associated with long‐term acid‐suppressive therapy. Vonoprazan (VPZ), a potassium‐competitive acid blocker, provides potent acid suppression and may induce hypergastrinemia, potentially contributing to the growth of hyperplastic polyps. However, clinical information regarding VPZ‐associated gastric hyperplastic polyps remains limited. We report three cases of severe bleeding from gastric hyperplastic polyps in patients who were administered VPZ. Case 1 involved a 92‐year‐old woman who presented with melena and anemia. Endoscopy revealed an 8‐cm hyperplastic polyp, and although resection was considered, it was deferred. After switching from VPZ to an H2‐receptor antagonist (H2RA), her anemia gradually improved, and the polyp regressed. Case 2 involved a 61‐year‐old man with hematemesis due to bleeding from multiple hyperplastic polyps. Emergency endoscopic mucosal resection was performed, and markedly elevated serum gastrin levels were noted. Following the replacement of VPZ with an H2RA, the size of the remaining polyps decreased. Case 3 involved a 54‐year‐old man with anemia and recurrent bleeding from hyperplastic polyps, requiring multiple endoscopic treatments. After VPZ discontinuation and H2RA initiation, the unresected polyps regressed. Two patients were undergoing hemodialysis, which may have exacerbated hypergastrinemia and bleeding. In all three cases, switching from VPZ to an H2RA resulted in the regression of gastric hyperplastic polyps and improvement of anemia. In patients receiving VPZ who develop progressive anemia or bleeding from hyperplastic polyps, discontinuation of VPZ or switching to an H2RA may be effective.

## 1 Introduction

Gastric hyperplastic polyps can cause gastrointestinal bleeding and may be associated with malignant transformation [1]. The long‐term use of proton pump inhibitors (PPIs) is known to induce enlargement of fundic gland polyps and hyperplastic polyps, which may lead to bleeding [1]. Vonoprazan (VPZ) is a potassium‐competitive acid blocker (P‐CAB) that has been available in Japan since 2015 and provides stronger acid suppression than PPIs. Similar to PPIs, VPZ‐induced enlargement of gastric polyps is a concern; however, available clinical information remains limited [1, 2, 3, 4]. We report three cases of bleeding from gastric hyperplastic polyps in patients taking VPZ, with no evidence of *Helicobacter pylori* infection (negative serum anti‐*H. pylori* antibodies).

## 2 Case Report

A total of three patients were admitted to Isesaki Municipal Hospital, Japan, between May 2024 and September 2025. Baseline characteristics and clinical details are summarized in Table 1. All three patients shared several clinical characteristics: they were receiving VPZ for treatment of gastroesophageal reflux disease, had hyperplastic polyps with bleeding in the upper stomach, had no history of *H. pylori* eradication therapy, showed no endoscopic evidence of gastric atrophy, and tested negative for serum anti– *H. pylori* antibodies, and no organisms suspicious for *H. pylori* were identified on hematoxylin–eosin staining of biopsy or endoscopic mucosal resection (EMR) specimens. In Cases 2 and 3, pathological examination of the background mucosa surrounding the hyperplastic polyps in the EMR specimens revealed findings suggestive of hypergastrinemia, including parietal cell protrusion and parietal cell hyperplasia. In Case 1, only biopsy specimens from the hyperplastic polyp were obtained, and therefore, pathological findings associated with hypergastrinemia could not be assessed.

**TABLE 1:** Details of the three patients with potassium‐competitive acid blocker‐associated gastric hyperplastic polyps with bleeding.

No.	Age	Sex	Comorbidities	Antiplatelet/antithrombotic drugs	Duration of VPZ administration (years)	PPI use prior to VPZ and duration of administration (years)	Location of the hyperplastic polyp with bleeding	Endoscopic atrophic change	Blood anti‐*Helicobacter pylori* antibody	Serum gastrin level (pg/mL)	Blood transfusion (units)
1	92	Female	GERD	(‐)	2	(+), Unknown	Upper	(‐)	(‐)	NA	10
2	61	Male	GERD, chronic renal failure, and hypertension	(‐)	1	(+), 11	Upper	(‐)	(‐)	2669	4
3	54	Male	GERD, chronic renal failure, and secondary hyperparathyroidism	(‐)	4	(‐)	Upper	(‐)	(‐)	NA	6

Abbreviations: GERD, gastroesophageal reflux disease; NA, not applicable; PPI, proton pump inhibitor; VPZ, vonoprazan.

### 2.1 Case 1

**FIGURE 1:**
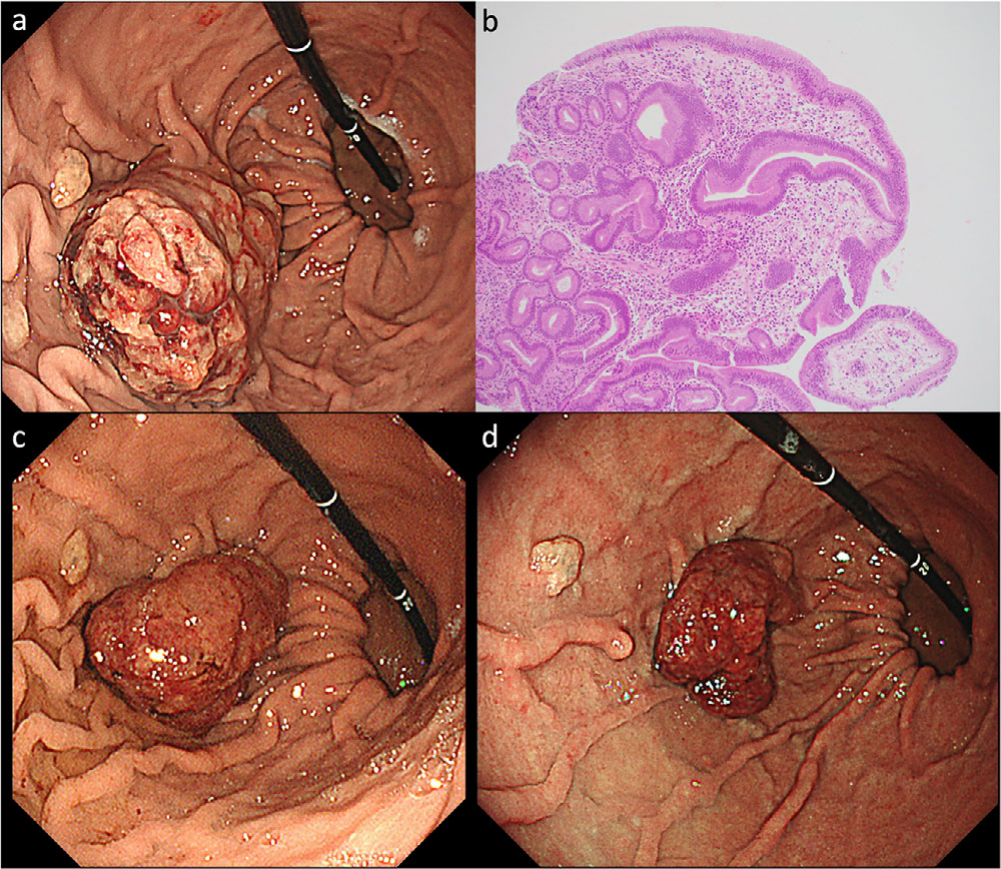
(a) Endoscopic image at diagnosis shows an 8‐cm lesion on the greater curvature of the upper gastric body. (b) Histopathological findings of the biopsy specimen (magnification × 100). Hyperplasia of the foveolar epithelium is observed within a loose, edematous stroma containing variable numbers of inflammatory cells. (c) Endoscopic image obtained 9 months after switching to H2‐receptor antagonist (H2RA), shows reduction of the hyperplastic polyp to 6 cm. (d) Endoscopic image obtained 15 months after switching to H2RA, shows further reduction of the hyperplastic polyp to 4 cm.

A 92‐year‐old woman presented with melena, dyspnea, and general fatigue. During clinical examination, anemia was identified. Esophagogastroduodenoscopy (EGD) revealed bleeding from a hyperplastic polyp located in the greater curvature of the upper gastric body. Although biopsies revealed no malignancy, the hyperplastic polyp was large (approximately 8 cm), and endoscopic resection was considered. However, the patient declined because of the invasive nature of the procedure. Conservative treatment was subsequently initiated. VPZ was switched to an H2‐receptor antagonist (H2RA) (famotidine 40 mg/day), and the patient was discharged. She required four units of red blood cells (RBCs) during hospitalization and an additional six units within 5 months after discharge. Her anemia gradually improved without further transfusion, and follow‐up endoscopy confirmed a reduction in the polyp size (Figure 1).

### 2.2 Case 2

**FIGURE 2:**
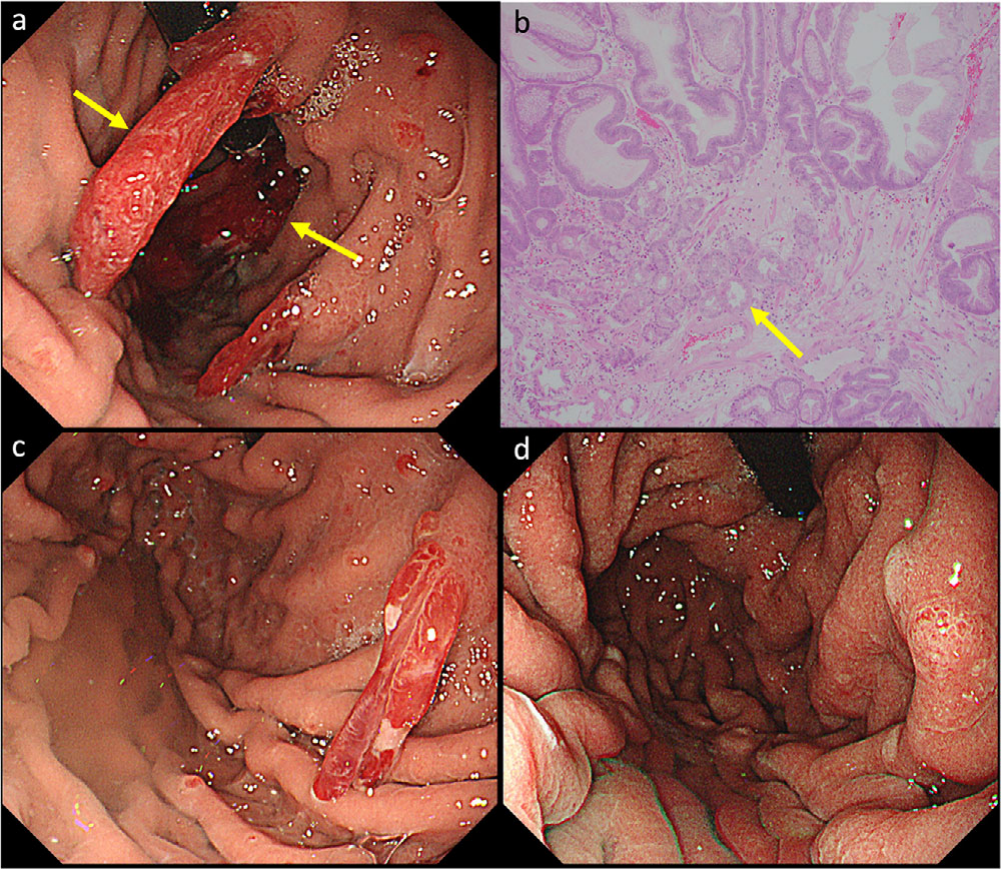
(a) Endoscopic image at diagnosis. The lesion indicated by the yellow arrow was resected by endoscopic mucosal resection (EMR). (b) Histopathological findings of the EMR specimen (magnification × 100). Parietal cell protrusion and parietal cell hyperplasia, which are indicated by the yellow arrow, are observed within a loose, edematous stroma containing variable numbers of inflammatory cells. (c) Endoscopic image obtained 1 month after EMR shows scarring at the resection site. d Endoscopic image obtained 7 months after switching to H2‐receptor antagonist (H2RA), shows reduction of the remaining hyperplastic polyps.

A 61‐year‐old man was admitted to the emergency department with hematemesis. Emergency EGD revealed bleeding from two hyperplastic polyps on the anterior wall of the upper gastric body, and EMR was performed. Pathological examination revealed no malignancy. Multiple additional hyperplastic polyps were also observed. Serum gastrin levels were markedly elevated (2668 pg/mL); therefore, polyp growth associated with VPZ was suspected. VPZ was replaced with famotidine (20 mg, three times weekly after hemodialysis), and the patient was discharged. He required four units of RBCs during hospitalization. Serum gastrin was re‐evaluated 6 months after switching from VPZ to an H2RA, and it had decreased to 58.3 pg/mL, and later follow‐up confirmed shrinkage of the unresected polyps (Figure 2).

### 2.3 Case 3

**FIGURE 3:**
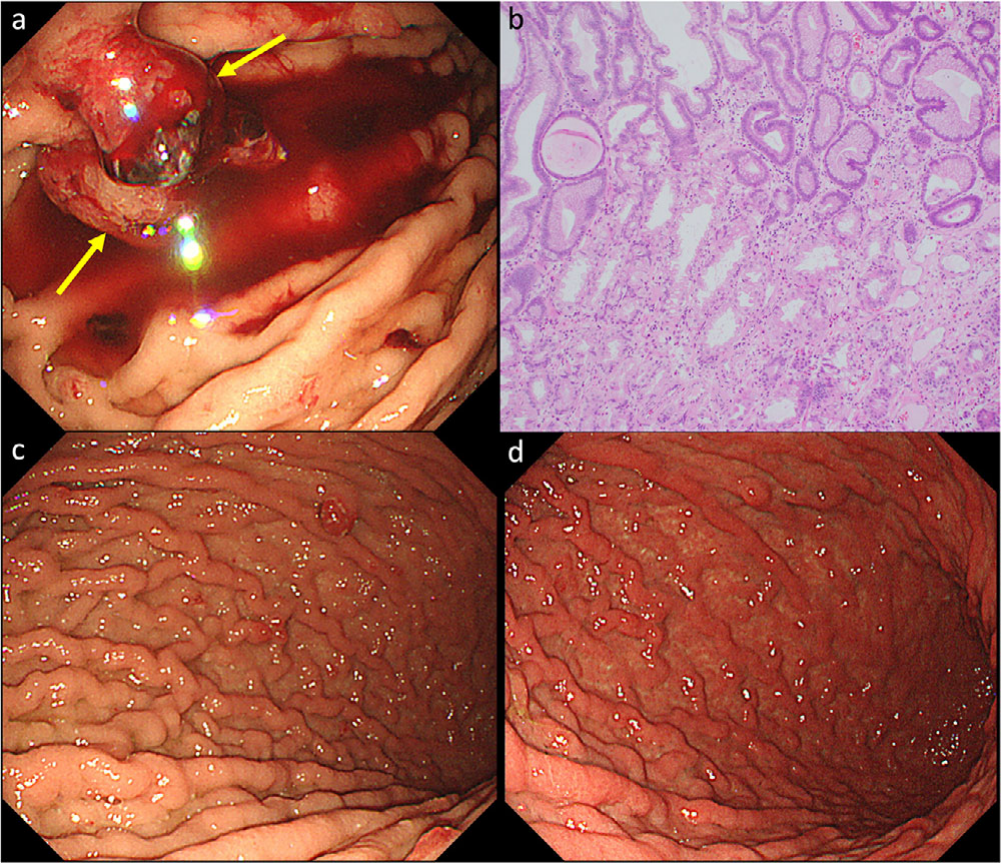
(a) Endoscopic image at diagnosis. The lesion indicated by the yellow arrow was resected by endoscopic mucosal resection (EMR). (b) Histopathological findings of the EMR specimen (magnification × 100). Parietal cell protrusion and parietal cell hyperplasia are observed. (c) Hyperplastic polyp that was not resected at the time of diagnosis. (d) Endoscopic image obtained 2 months after switching to famotidine shows a reduction of the unresected hyperplastic polyp.

A 54‐year‐old man underwent EGD for dizziness and anemia at a referring hospital. The EGD revealed bleeding from a hyperplastic polyp. The patient was referred to our hospital, where an EMR was performed for a bleeding polyp on the posterior wall of the upper gastric body. Two days later, the patient experienced recurrent hematemesis due to bleeding from another polyp on the anterior wall of the upper gastric body. Hemostasis was achieved using hemostatic forceps, followed by EMR of the two lesions. No malignancy was observed upon histological examination. VPZ was changed to famotidine (20 mg, three times weekly after hemodialysis), and the patient was discharged. He required six units of RBCs during hospitalization. Subsequent follow‐up revealed shrinkage of the residual polyps (Figure 3).

## 3 Discussion

In all cases, switching from VPZ to H2RA resulted in the shrinkage of the hyperplastic polyps, suggesting that discontinuation of VPZ may be an effective strategy for controlling bleeding. All three patients had been taking VPZ for reflux esophagitis, had no evidence of *H. pylori* infection, and had experienced bleeding from polyps located in the upper stomach.

In Japan, available acid‐suppressive agents include H2RAs, PPIs, and P‐CABs. Long‐term P‐CAB therapy is generally reserved for patients requiring potent acid suppression. Although the patients did not undergo multiple *H. pylori* diagnostic tests and therefore did not strictly meet the definition of uninfected status [5], they showed no endoscopic evidence of gastric atrophy, and all had negative serum *H. pylori* antibodies, suggesting preserved gastric acid secretion. In routine clinical practice, when endoscopic findings do not suggest *H. pylori* infection, additional diagnostic tests are not routinely performed. However, because eradication therapy could potentially induce regression of hyperplastic polyps if infection were present, serum anti‐*H. pylori* antibodies were measured to confirm negativity. Consequently, PPI‐based symptom control was inadequate, necessitating the use of VPZ.

Long‐term PPI and VPZ therapies are significantly associated with the development of hyperplastic polyps [1]. Hypergastrinemia and increased expression of gastrin receptors in the foveolar epithelium are believed to contribute to polyp formation [6]. Furthermore, a positive correlation was observed between serum gastrin levels and hyperplastic polyp development [7]. Although in this case series hypergastrinemia was not confirmed in all cases, VPZ has also been reported to induce significantly higher levels of serum gastrin than PPIs [5], suggesting a potentially higher risk of polyp growth. Additionally, some patients may have received PPI therapy before starting VPZ, further increasing the cumulative exposure. Furthermore, two of the three patients had end‐stage renal disease and were on hemodialysis. Impaired gastrin metabolism due to renal dysfunction may have worsened hypergastrinemia [8], and the use of heparin during dialysis may have contributed to the enlargement and bleeding of the hyperplastic polyps.

Although fundic gland polyps associated with PPI or VPZ therapy are known to shrink within approximately 2 months after discontinuation [3, 9], transfusion requirements in some patients in this series persisted for up to 5 months, suggesting that regression of hyperplastic polyps may take longer than that of fundic gland polyps. Therefore, endoscopic resection may be necessary in cases where anemia is difficult to control.

In this case series, no evidence of active or prior *H. pylori* infection or malignant transformation was detected in any of the hyperplastic polyps. To our knowledge, there are no reports of cancer arising within hyperplastic polyps in *H. pylori*‐negative patients taking PPIs or P‐CABs; only two cases have been reported in *H. pylori*‐negative patients without PPI and P‐CAB exposure [10]. Although hyperplastic polyps have been considered precancerous in some settings, this risk appears to be primarily based on data from patients with prior *H. pylori* infection, and the risk profiles of uninfected patients may differ.

In conclusion, in patients receiving VPZ who develop progressive anemia due to bleeding from gastric hyperplastic polyps, discontinuation of VPZ or switching to H2RA may be effective.

## Author Contributions

Kengo Kasuga prepared the first draft of this manuscript. Sakuya Katakai, Shimizu Megumi, Ayaki Isshiki, Shingo Ishihara, Xing Hua Ma, and Takashige Masuo managed the patients. Hiroomi Ogawa, Yoji Takeushi, and Toshio Uraoka revised the manuscript. All the authors approved the final version of the manuscript.

## Funding

The authors received no specific funding for this work.

## Ethics Statement

All procedures followed were in accordance with the ethical standards of the responsible committee on human experimentation (institutional and national) and with the Helsinki Declaration of 1975, as revised in 2008.

## Consent

Informed consent was obtained from the patients for publication of the report and associated images.

## Conflicts of Interest

Toshio Uraoka is one of the Deputy Editors‐in‐Chief of the DEN Open. Yoji Takeuchi is an Associate Editor of DEN Open. The other authors declare no conflicts of interest.
